# Beyond Carrier Status: *CFTR* Heterozygosity as an Overlooked Clinical Risk Factor for Pancreatitis

**DOI:** 10.1111/cge.14780

**Published:** 2025-06-05

**Authors:** Lucas D. Richter, Douglas M. Ruderfer, Josh F. Peterson, Lisa A. Bastarache

**Affiliations:** ^1^ Division of Genetic Medicine, Department of Medicine Vanderbilt University Medical Center Nashville Tennessee USA; ^2^ Vanderbilt Genetics Institute Vanderbilt University Medical Center Nashville Tennessee USA; ^3^ Department of Biomedical Informatics Vanderbilt University Medical Center Nashville Tennessee USA; ^4^ Department of Psychiatry & Behavioral Sciences Vanderbilt University Medical Center Nashville Tennessee USA; ^5^ Division of General Internal Medicine, Department of Medicine Vanderbilt University Medical Center Nashville Tennessee USA

**Keywords:** *CFTR* heterozygosity, cystic fibrosis‐related phenotypes, genetic risk factors, pancreatitis, phenome‐wide association study (PheWas), precision medicine, prenatal carrier screening

## Abstract

This study assessed the effect of *CFTR* pathogenic variant status, detected during prenatal carrier screening, for the incidence and clinical recognition of cystic fibrosis‐related phenotypes. Data were queried from the Vanderbilt University Medical Center clinical genetic database (CGdb), which includes clinically reported pathogenic variants and electronic health records (EHRs) from 2001 to 2023. Based on carrier screening results, we identified individuals heterozygous for a pathogenic *CFTR* variant and those who tested negative. Logistic regression tested associations between *CFTR* carrier status and 11 cystic fibrosis (CF)‐related phenotypes. A phenome‐wide association study (PheWAS) was performed to identify additional phenotypic associations, and manual chart review was conducted to evaluate recognition and clinical application of *CFTR* carrier status in patients diagnosed with pancreatitis. Among 12,082 women tested, *CFTR* carriers (*n* = 451) were at significantly higher risk of developing acute pancreatitis (*p* = 3.93 × 10^−6^; OR = 4.68 [2.43–9.00]). No other CF‐related phenotypes were significantly associated in this female cohort. Manual chart review revealed that *CFTR* carrier screening results were not clinically correlated with pancreatitis diagnoses. In this large cohort of women tested for prenatal carrier screening, *CFTR* pathogenic variants relevant to pancreatitis were overlooked, despite informing etiology, management, and prognosis.

## Graphical abstract

*CFTR* carrier status detected via prenatal screening is associated with increased pancreatitis risk in women, but clinical recognition remains low. Integrating carrier results into electronic health records may enhance personalized care and aid in early diagnosis.

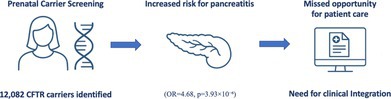

## 1 Introduction

Cystic fibrosis (CF) is a genetic disorder caused by variants in the *CFTR* gene, affecting multiple organ systems, including the lungs, pancreas, and digestive tract. CF manifests when an individual inherits two pathogenic variants in *CFTR*. While individuals carrying a single pathogenic *CFTR* variant do not develop classical CF, numerous population‐based studies suggest a modestly elevated risk of CF‐related phenotypes—most consistently pancreatitis—while effect sizes for respiratory conditions are small and sometimes nonsignificant.

Understanding of *CFTR* carrier risks has evolved [1]. Early studies found no significant differences between *CFTR* carriers and controls [2]. However, as sample sizes grew, risk factors began to emerge [3]. For example, in a study of over 1000 *CFTR* carriers, respiratory infections including sinusitis, bronchitis, and pneumonia were more common [4]. The largest study to date, involving nearly 20,000 *CFTR* carriers, found increased risk for 56 CF‐related phenotypes across multiple organ systems, including the bone, endocrine, respiratory, digestive, and reproductive systems [5]. Additionally, *CFTR* heterozygosity has been shown to be a strong risk factor for chronic pancreatitis (CP), with one study estimating that *CFTR* contributed to 24% of idiopathic CP cases [6]. Many additional studies have linked carrier status to CF‐related phenotypes, though reported risk profiles vary [4, 5, 7].

Despite extensive evidence suggesting *CFTR* carriers are at increased risk for certain conditions, clinical significance for individuals remains questionable. *CFTR* heterozygosity is a reported risk factor when discovered through genetic testing for pancreatitis. However, patients identified as *CFTR* carriers through non‐diagnostic testing, such as prenatal carrier screening, are not typically informed of risk [8, 9], Hence, the personal health implications for *CFTR* carriers identified through routine prenatal screening remain poorly understood. More studies are needed that examine clinical relevance in screened patients.

This study examines the personal health implications of *CFTR* carrier status using a cohort of over 12,000 women who underwent carrier screening. We address two key questions: (1) Are the *CFTR*‐related phenotypes identified in previous studies more prevalent among women found to be *CFTR* carriers through prenatal screening? (2) How do clinicians utilize this information? Notably, because *CFTR* carrier status is identified outside a diagnostic context, it is unclear if this information is typically correlated with patient symptoms. Our findings indicate that *CFTR* carriers identified via screening are more likely to have pancreatitis. However, clinicians often do not correlate these phenotypes to the *CFTR* genotype, even among symptomatic patients.

## 2 Methods

### 2.1 Data Used in This Study

Data was drawn from the clinical genetic database (CGdb), a database of clinical genetic tests extracted from electronic health records (EHRs) [10]. Included patients received prenatal screening for *CFTR*, either as a single gene or panel, from 2001 to 2023. Patients with a single pathogenic *CFTR* variant were considered carriers. We excluded patients with conflicting or biallelic variants [11]. Isolated findings of poly‐T tract polymorphisms were treated as benign. *CFTR* variants were annotated as residual versus minimal function (Table S1) [12]. Carrier screening tests and International Classification of Diseases (ICD) codes were restricted to those that occurred after age 15. Visit days were defined using ICD entry dates; the visit years variable was defined as the number of unique years a patient visited VUMC. ICD codes were translated to phecodeX [13].

### 2.2 Testing for Associations With Known *CFTR* ‐Related Phenotypes

We attempted replication of the findings of Miller et al. in our cohort, using their reported 56 CF‐related phenotypes as targets of our analysis. We mapped 43 of these phenotypes to phecodeX, excluding those lacking relevance or a suitable phecode representation (Table S2). Prevalences of the CF‐related phenotypes were computed for carriers and unaffected controls. For respiratory phenotypes, we performed power calculations for detecting odds ratios (OR) of 1.5 and 2.0, and those from Miller et al. using a significance level (alpha) of 0.05 and assuming a two‐sided test. Using logistic regression, we tested for association between *CFTR* carrier status and target CF‐related phenotypes with at least 40 cases, using the phecode as the independent variable and carrier status as the main dependent variable. We included covariates for age at last visit, last year, EHR‐defined race/ethnicity, and number of visit years. To detect associations beyond those included in the targeted analysis, we conducted a phenome‐wide association (PheWAS) of *CFTR* carrier status using the same logistic regression model described above.

### 2.3 Assessing the Clinical Use of *CFTR* Carrier Status in Affected Individuals

Chart review assessed whether clinicians linked carrier status to pancreatitis. Manual review was conducted by a genetic counselor, and information extracted included dates of pancreatitis diagnoses and whether *CFTR* carrier status was mentioned as a potential underlying cause.

## 3 Results

### 3.1 Cohort

The analysis included 12 082 women who underwent carrier screening, of which 451 carried a pathogenic *CFTR* variant. (Table 1.) We observed a carrier frequency of 3.73%, which is modestly higher than previously reported for North American populations [14]. The majority of the variants (*n* = 385; 85.4%) were minimal function, while the remainder were residual function (*n* = 67; 14.9%).

**TABLE 1:** Demographic information and mean follow up time for patients who underwent carrier screening.

Metric	Value
*N*	12 082
Age (mean ± SD)	34.42 ± 8.06
Year of follow‐up (mean ± SD)	7.39 ± 5.27
EHR race/ethnicity	
White	8397 (69.5%)
Black	1605 (13.3%)
Hispanic	1006 (8.3%)
Asian	379 (3.1%)
Multiple/other	392 (3.2%)
Unknown	303 (2.5%)
*CFTR* carriers	451 (3.7%)

### 3.2 Underutilization and Misuse of ICD Codes Indicating *CFTR* Carrier Status

Only 253 *CFTR* carriers (56.1%) received an ICD code indicating *CFTR* carrier status (ICD‐9 V83.81 or ICD‐10 Z14.1). Additionally, 22 patients with negative screening tests had an ICD for *CFTR* carrier status, 10 of whom tested positive for a poly‐T tract polymorphism alone, and the remainder whose screening tests were entirely negative.

### 3.3 Targeted Association Results

**FIGURE 1:**
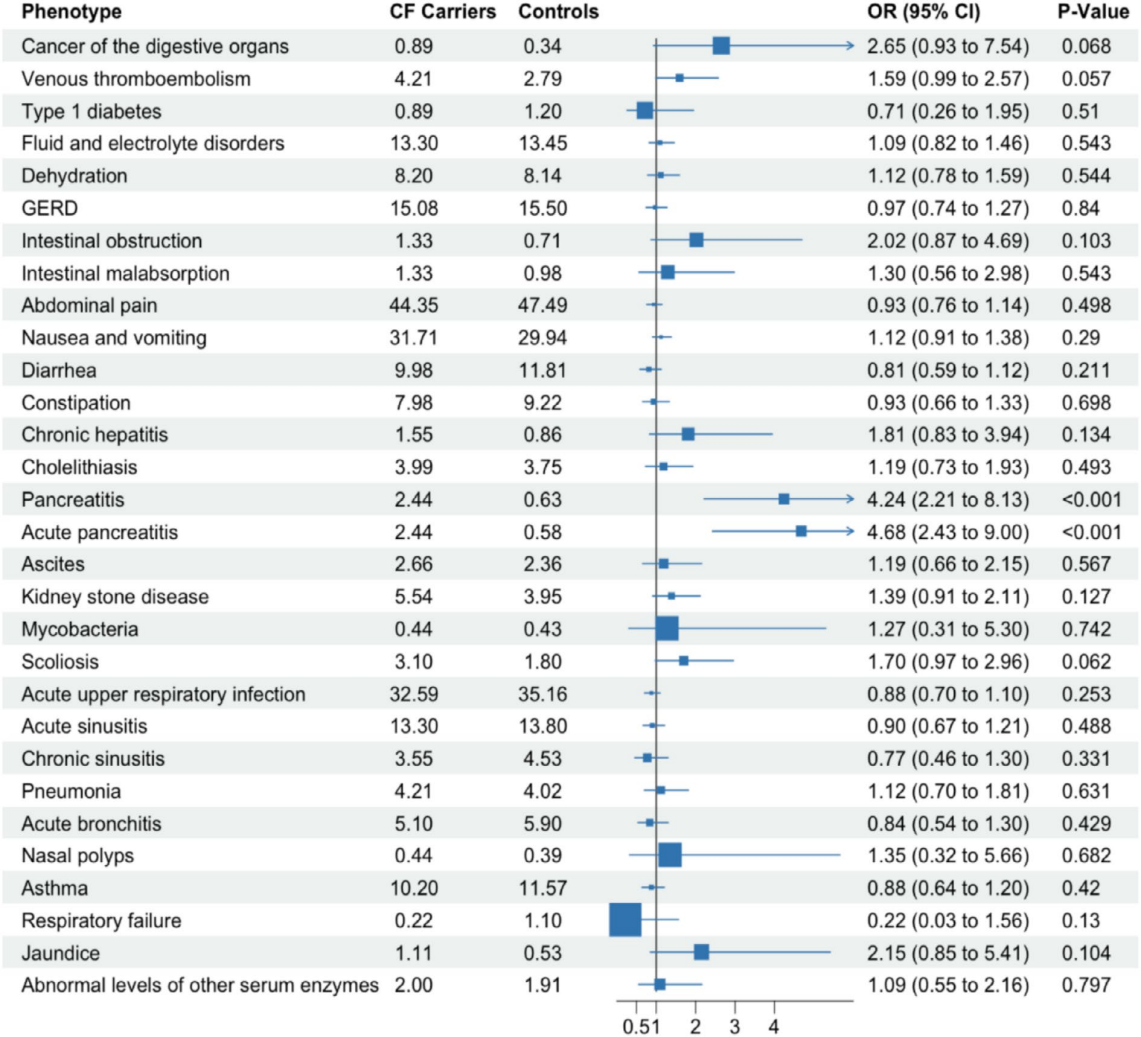
Forest plot comparing the likelihood of CF‐related phenotypes between CF carriers and controls. The *x*‐axis displays odds ratios (ORs) with 95% confidence intervals, and corresponding *p*‐values. An OR > 1 indicates increased odds of the phenotype among CF carriers relative to controls. Notably, pancreatitis and acute pancreatitis show significantly elevated odds in CF carriers (*p* < 0.001).

24 of 43 CF‐related phenotypes had an elevated prevalence among *CFTR* carriers (Table S3). The targeted logistic regression analysis revealed two statistically significant phenotypes: Acute pancreatitis (phecode GI_554.11) and Pancreatitis (GI_554.1, the parent phecode of GI_554.11). None of the eight respiratory phenotypes were statistically significant. (Figure 1). Six of eight respiratory phenotypes were powered at ≥ 80% to detect OR ≥ 2; all were underpowered to detect ORs reported in Miller et al. (Table S4). In a PheWAS of 1130 phenotypes, only pancreatitis and acute pancreatitis were significant following Bonferroni correction (Table S5).

### 3.4 Assessing for Clinical Correlations Among *CFTR* Carriers With Pancreatitis

Acute pancreatitis was rare in the cohort, with a prevalence of 2.44% among *CFTR* carriers and 0.63% among controls. However, the likelihood of being a *CFTR* carrier given an acute pancreatitis diagnosis was quite high; nearly one in seven patients with pancreatitis (14.1%). Among the 11 *CFTR* carriers who developed pancreatitis, one (9.1%) carried a residual function variant, and 10 (91%) had minimal function variants vs. 14.9% of the overall cohort; this difference was not statistically significant (Fisher's exact test, *p* > 0.05). Chart review corroborated all pancreatitis diagnoses in *CFTR* carriers and demonstrated that carrier screening results were never used for diagnostic refinement. Seven patients had confirmed pathogenic *CFTR* variants prior to their pancreatitis diagnosis with a mean of 5 years from genetic testing to pancreatitis diagnosis. Four patients received *CFTR* testing after their pancreatitis diagnosis: two following pancreatitis panels yielding only monoallelic *CFTR* variants, which led to expanded carrier screening for family planning; one with first‐trimester pancreatitis, screened within 1 month; and one 12 years after initial pancreatitis. Only the two patients tested after a pancreatitis panel had clinical recognition of *CFTR*‐mediated risk. Of the six patients with noted etiological basis for pancreatitis, three had gallstone‐related, and three post‐Endoscopic retrograde cholangiopancreatography (ERCP) pancreatitis.

## 4 Discussion

In this study, we found that women identified as *CFTR* carriers through prenatal carrier screening have a significantly higher risk of developing pancreatitis. This result aligns with previous studies linking *CFTR* heterozygosity to pancreatitis [1, 3, 5, 6]. Our study has several strengths. First, we identified *CFTR* carriers using clinical genetic testing, ensuring the accuracy of our genotyping data. Second, our study was conducted in a real‐world clinical population, allowing us to evaluate the clinical significance of *CFTR* carrier status in a practical, real‐world scenario.

Our review of patient records indicates that the connection between carrier status and pancreatitis diagnosis is typically overlooked. This oversight is likely due, in part, to the way genetic test results are stored in the EHR. In most cases, carrier screening results were saved as PDF documents, making them difficult to search and integrate into clinical workflows [10]. Additionally, pancreatitis diagnoses often occurred years after the *CFTR* carrier status was first documented, further complicating the recognition of this connection.

We did not observe an increased risk for respiratory conditions, which have been observed even in studies based on smaller cohorts. Several factors may explain this discrepancy. First, while we were powered to detect associations for six respiratory phenotypes with moderate effect sizes, we were underpowered to detect smaller effect sizes reported in larger studies, including Miller et al. Second, most prior studies identified *CFTR* carriers using ICD claims data or inferred carrier status based on CF diagnoses in first‐degree relatives. These methods may introduce bias through undetected compound heterozygosity, ascertainment bias from the selective detection of *CFTR* variants in a population, or misclassification due to incorrect ICD codes (as observed in this study). Third, previous studies were based on heterogeneous cohorts, encompassing pediatric and adult patients. Our study focused on women of reproductive age. These cohort differences may influence the relationship between *CFTR* carrier status and CF‐related phenotypes.

Additionally, our study highlights a potential path for future research by leveraging clinical genetic testing data to explore genotype–phenotype relationships without requiring new genotype data from research settings. As clinical genetic data continues to accumulate in electronic health records, multi‐institutional collaborations and integration with national biobanks could improve power to assess lower‐penetrance *CFTR*‐related outcomes.

Although carrier screening tests are not intended for diagnostic purposes, our findings suggest that these results may have significant personal health implications. While only a small proportion of *CFTR* carriers develop pancreatitis, a significant subset of patients with pancreatitis were found to be *CFTR* carriers. While routine follow‐up may not be necessary for every *CFTR* carrier, a system that alerts clinicians to a patient's *CFTR* carrier status could be highly valuable when pancreatitis is diagnosed or suspected. Implementing EHR infrastructure that organizes and stores genetic test results would allow for more effective clinical utilization, ensuring that *CFTR* carrier status is readily available—and clinically actionable—if patients present with relevant symptoms. However, barriers to implementation remain. Most clinical genetic test results are currently stored in unstructured, non‐computable formats, limiting their accessibility for clinical decision support [10]. Additionally, clinician awareness of actionable findings may be limited, and variability across EHR systems complicates the integration of structured genetic data into clinical workflows. This study highlights one use‐case among many for how integrating genetic test results into the EHR could improve patient care.

Integrating genetic data into clinical workflows may enhance precision medicine and improve outcomes for individuals with *CFTR* variants [15, 16]. Identifying pathogenic *CFTR* variants in patients with pancreatitis clarifies etiology, enables timely diagnosis, and guides management—potentially including *CFTR* modulators (e.g., ivacaftor), which are currently only approved for patients with formal diagnoses of CF. However, preliminary reports suggest potential benefit for patients with *CFTR*‐related pancreatitis, but reported benefits and risks are inconsistent [17, 18, 19]. Overlooking *CFTR* status may lead to diagnostic delays, misaligned treatments, and potentially unnecessary procedures such as ERCP, which can increase complications or even trigger pancreatitis [1, 7]. Making genetic carrier status searchable in EHRs could enable clinicians to implement proactive screening and prevention strategies, such as promoting lifestyle changes to reduce pancreatitis risk or monitoring for gastrointestinal cancers.

This study has limitations. First, it was conducted in a single academic medical center. To establish the generalizability of our findings, external replication is needed. Second, because our cohort is relatively young, we may have missed significant associations with CF‐related phenotypes that occur later in life, such as degenerative pulmonary diseases and cancers. Indeed, we found digestive system cancers were 2.7 times higher in *CFTR* carriers than controls, echoing a finding reported in a UK biobank study of older *CFTR* carriers—although not significant in the 44 cases analyzed here [20]. Additional longitudinal data is needed to fully evaluate later onset conditions and phenotypes that might present more commonly in men—not represented here. Third, *CFTR* testing in our cohort was primarily performed with limited panels, consistent with standard practice at the time. Therefore, we cannot exclude the possibility of undetected second *CFTR* variants, modifier alleles, or other pancreatitis‐associated gene variants (i.e., *SPINK1*, *PRSS1*, or *CTRC*). Finally, while our cohort was larger than many previous studies of *CFTR* carriers, we were underpowered to detect associations for rare phenotypes or small effect sizes.

## 5 Conclusion

This study highlights the clinical significance of *CFTR* carrier status that is incidentally discovered through carrier screening, pointing out a potential missed opportunity to correlate these genetic findings with clinical diagnoses. It also illustrates the differences between research observations and real‐world clinical scenarios, emphasizing the potential to leverage clinical genetic testing data to enhance patient care.

## Author Contributions

**L.A.B.:** conceptualization. **L.A.B. and L.D.R.:** data curation. **L.A.B.:** formal analysis. **L.A.B., L.D.R.:** investigation. **L.A.B. and L.D.R.:** methodology. **L.A.B.:** visualization. **L.A.B. and L.D.R.:** writing – original draft. **L.A.B., L.D.R., D.M.R., and J.F.P:** writing – review and editing. **L.A.B.:** supervision.

## Ethics Statement

This study was reviewed by the Vanderbilt University Medical Center IRB 171011. All clinical data were de‐identified prior to use.

## Conflicts of Interest

L.D.R. and J.F.P. declare no conflicts of interest. L.A.B. receives royalties from Nashville Biosciences, and D.M.R. has served on advisory boards for Illumina and Alkermes and has received research funds unrelated to this work from PTC Therapeutics and Sanofi.

## Peer Review

The peer review history for this article is available at https://www.webofscience.com/api/gateway/wos/peer‐review/10.1111/cge.14780.

## Supporting information


**Data S1.** Supporting Information.

## Data Availability

Summary level data regarding significant phenotypes from logistic regression and PheWas are presented in the results section and in Figure 1. Summary data on clinical and genetic information are provided throughout the paper. All requests for raw (e.g., *CFTR* variants and phenotype) data and materials are reviewed by Vanderbilt University Medical Center to determine whether the request is subject to any intellectual property or confidentiality obligations. For example, patient‐related data not included in the paper may be subject to patient confidentiality. Any such data and materials that can be shared will be released via a material transfer agreement.
